# Association of medial arch support of foot orthoses with knee valgus angle at initial contact during cutting maneuvers in female athletes: a controlled laboratory study

**DOI:** 10.1186/s13102-022-00608-w

**Published:** 2022-12-19

**Authors:** Seikai Toyooka, Keisuke Tsukada, Youich Yasui, Yasuaki Saho, Yasuaki Okawa, Shuji Ando, Takumi Nakagawa, Hirotaka Kawano, Wataru Miyamoto

**Affiliations:** 1 Department of Orthopaedic Surgery, Teikyo University, Tokyo, Japan; 2 Tokyo University of Science, Tokyo, Japan; 3 Faculty of Medical Technology, Teikyo University, Tokyo, Japan

**Keywords:** Foot orthosis, Medial arch support, Knee valgus angle, Cutting manoeuvres

## Abstract

**Background:**

The effect of medial arch support foot orthoses on kinematics and kinetics of the knee joint has remained unknown.

**Methods:**

Sixteen female collegiate-level athletes volunteered to participate. Participants were asked to perform a 30° sidestep cut using orthoses of 3 different medial arch heights, comprising of the following: (1) “low,” a full flat foot orthosis without arch support, (2) “mid,” a commercially available foot orthosis with general height arch support, and (3) “high,” a foot orthosis with double the commercially available height for arch support to observe the effect on the knee when overcorrected. Kinematics and kinetics of the knee joint were collected by a markerless motion capture system with 2 force plates and compared between orthosis types using linear regression analysis, assuming a correlation between the measurements of the same cases in the error term.

**Results:**

The knee valgus angle at initial contact was 2.3 ± 5.2 degrees for “low” medial arch support height, 2.1 ± 5.8 degrees for “mid,” and 0.4 ± 6.6 degrees for “high”. Increased arch support height significantly decreased the knee valgus angle at initial contact (*p* = 0.002). Other kinematic and kinetic measurements did not differ between groups.

**Conclusions:**

The valgus angle of the knee at initial contact was decreased by the height of the medial arch support provided by foot orthosis during cutting manoeuvres. Increasing the arch support height may decrease knee valgus angle at initial contact. Medial arch support of foot orthosis may be effective in risk reduction of ACL injury.

*Clinical trial registration numbers and date of registration*: UMIN000046071, 15/11/2021.

## Introduction

Anterior cruciate ligament (ACL) tears account for over half of knee injuries, affecting more than 200,000 knee joints each year in the United States with direct and indirect costs that exceed $7 billion annually [1, 2]. Left untreated, complete ACL tears can cause knee instability, damage to the meniscus and cartilage surfaces, osteoarthritis, and other pathological knee conditions [3]. At least nine months of rehabilitation and training are generally required to return to sport after ACL surgery [4–6]; therefore, the risk reduction of ACL tears is important for preventing osteoarthritis but also crucial for athletes to maintain and achieve a high level of performance.

Because of intrinsic factors such as increased quadriceps angle and increased posterior tibial slope, females are more susceptible to ACL injuries [7]. Although damage can also occur during rapid deceleration and landing, it occurs mainly during a cutting manoeuvre that requires a lateral change in direction [8, 9]. In non-contact injuries, knee valgus and internal rotation of the lower leg have been reported to occur at approximately 40 ms after the initial contact, at which time the ACL is injured [10–12]. During this combination of motions, a greater valgus angle of the knee and internal rotation moment of the lower leg result in a greater risk of injury. Although neuromuscular training programs have been developed and have been shown in some studies to reduce risk of injury, methods to prevent these abnormal knee movements may benefit from further refinement [13–19].

Although foot orthoses are indicated for a variety of foot and lower limb problems to reduce pain and improve functional performance, several studies have described the use of foot orthoses as a potentially effective solution for the risk reduction of ACL injuries [20–22]. Jenkins et al. [21] reported that female collegiate basketball players without foot orthoses were 7.14 times more likely to sustain an ACL injury than those with foot orthoses. This study speculated that the medial arch may decrease the hindfoot valgus angle, provide correction of knee valgus, and result in less ACL injury. However, little research has been conducted on analysing these movements in sports with the use of foot orthoses. To our knowledge, there are no previous reports on the motion analysis of the cutting motion with a particular focus on foot orthoses, which is known as the motion most likely to cause ACL injury.

The purpose of the present study was to clarify the effect of a medial arch support foot orthosis on kinematics and kinetics of the knee joint in female athletes. The result of this study may suggest the efficacy of foot orthosis in risk reduction of ACL injury and its usefulness in rehabilitating patients after ACL surgery. Therefore, the objective of this controlled laboratory study was to determine whether medial arch support of foot orthoses can prevent the increase of knee valgus angle and knee valgus moment during cutting manoeuvres in female athletes.

## Materials and methods

### Design

**Fig. 1:**
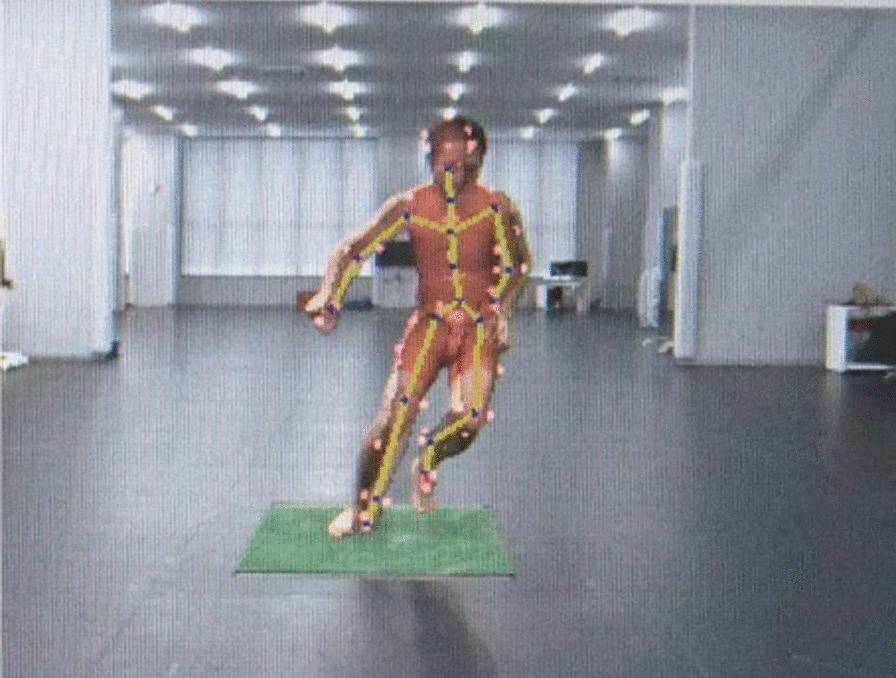
Photograph of the cutting manoeuvre

The experimental design was based on an established method described by Dowling et al. [23]. Light-coloured and close-fitting clothing was used for the investigation to allow an easier identification of the body surface. In a laboratory setting, participants were asked to perform a sidestep cut of 30° from their dominant leg with 3 different medial arch height orthoses. The sidestep cut manoeuvre, angled at 30° from the direction of travel, is a commonly used criterion in study designs that evaluate the risk of ACL injury [24–27]. When participants were asked to cut off from their dominant leg on a force plate, the 30° angle was first marked by tape on the floor of the laboratory to step off from the same place and direction. The footprint of the cut-off point was subsequently marked by tape on the force plate. A space of 20 m in front of the cutting point and 10 m at the back was secured to allow for cutting manoeuvre at sufficient speed in laboratory (Fig. 1). Participants were asked to practice the task until they could cut off from the predefined marks and were instructed to self-select the starting position and foot with which to perform their cutting task.

### Participants, recruitment, and sampling strategies

Sixteen female participants volunteered for this investigation. The characteristics of participants are described in Table 1. Of the 16 participants, 9 played collegiate level basketball and 7 played collegiate volleyball. These subjects were without (1) a history of musculoskeletal injuries requiring surgery, (2) current symptoms of pain, and (3) current injury of the lower limb. None of the subjects received training regarding ACL protection before or during the experiment.

**Table 1:** Subject characteristics

Number of subjects	16
Mean age	20.1 ± 0.7
Mean height (cm)	161.0 ± 5.7
Mean weight (kg)	53.5 ± 5.0
Mean BMI	20.6 ± 1.0
Sprots	Basketball: 9
	Valley ball: 7
Dominant side	Right: 16

The nature and purpose of the research, probable risks and benefits, and alternative treatment options were disclosed to the patients. Informed written consent was obtained from all participants prior to data collection and approved by the institutional review board. The height, weight, and mass of patients were measured and recorded after informed consent was obtained. Measurements were performed in a controlled environment with an attached clinic in case of a need for immediate care.

### Intervention

**Fig. 2:**
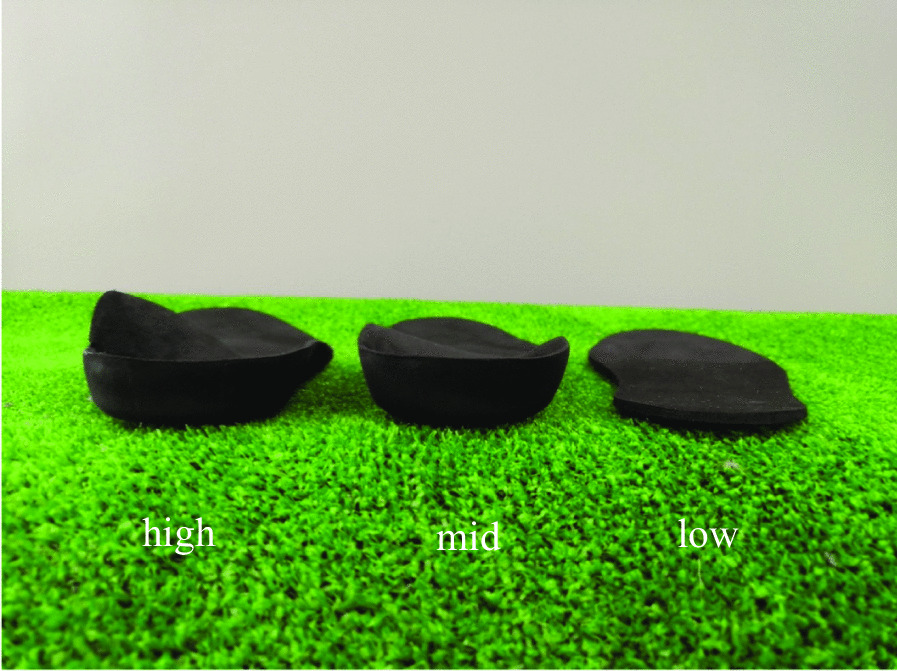
Three medial arch support heights of the foot orthoses

**Fig. 3:**
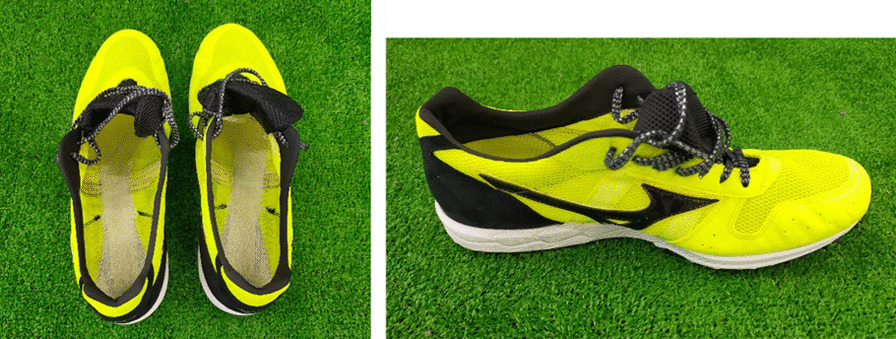
The running shoe with a thin sole

Three foot orthoses with different medial arch support heights were used in this investigation. In order to standardize the materials used for the foot orthoses used in this study, all foot orthoses were made from the same materials by an assembly technician (Fig. 2). First, a full flat foot orthosis without medial arch support was defined as “low.” Second, a foot orthosis with general height arch support comparable to commercially available foot orthoses (Footcraft®, Nippon Sigmax Co., Ltd., Tokyo, Japan) was defined as “mid.” Third, a foot orthosis with double the general height arch support of commercially available foot orthoses was defined as “high” to observe the effect on the knee when overcorrected. In the reference foot orthosis, the peak of the arch was at 65% from the front. The height of the top of the arch was approximately 1 in. with a selected size of 9 in. Each type of foot orthosis was created in four sizes that were scalable to all common women’s shoe sizes. The material was ethylene vinyl acetate with a hardness of 70 (SHORE 00 scale). To minimize the effect of the shoe sole on lower limb kinematics instead of basketball and volleyball shoes, a shoe with the thinnest available sole (Wave Cruise Japan, Mizuno Corporation, Osaka, Japan) was selected from 5 global sports shoe brands (Fig. 3). As with the foot orthoses used in this study, running shoes were also prepared in four sizes in 1-in. increments between 8 and 11 in.

The order of the type of foot orthoses worn by each participant was randomly selected for investigation. Prior to recording data, participants were allowed to practice on each foot orthosis and perform multiple cutting tasks to establish the fastest-comfortable speed while using the low arch foot orthosis based on the method described by Dowling et al. [23]. Once this speed was determined by the participant, it was then used as the standard running speed for all conditions. Three acceptable trials were completed for the cutting task on each foot orthosis. In order to avoid fatigue, participants rested for 1 min between each trial. An acceptable trial was defined as a completion of the task within 0.3 m/s of the standard running speed that was recorded without missing data.

### Data collection

Full body kinematic and lower limb kinetic data were collected by a markerless motion capture system combined with 2 force plates. Eight synchronized VGA colour cameras with a 2-megapixel resolution (Matrix Vision GmbH, Oppenweiler, Germany) were used in conjunction with a multi-channel data acquisition system (Simi Reality Motion Systems GmbH, Unterschleissheim, Germany) at a capture rate of 120 Hz to create the video recordings of the trials. A visual hull was constructed from silhouette images at every frame using a previously described shape-from-silhouette technique [28]. Subject-specific models of participants were generated with a full-body laser scan (Cyberware, Monterey, California) that automatically constructed scanned models. These models consisted of 15 rigid segments with 6 degrees of freedom between adjacent segments to identify the joint centers between body segments [29]. The full-body model was then matched to the visual hulls using a previously described matching process [30]. The matching was conducted during the entire recorded sequence to locate the joint centres of the subject. Two multi-component force plates (Bertec, Columbus, Ohio) were used at a frequency of 120 Hz and synchronized to the video feeds in order to record the ground reaction forces and moments.

Following the identification of the joint centres for the entire sequence, the kinematic and kinetic data were calculated based on a previously described method [31]. The primary outcome of this study was the knee valgus angle, which was evaluated twice. The first knee valgus angle was evaluated at initial contact, and the second was evaluated at peak ground reaction with the force plate during the stance phase. The stance phase of the cutting manoeuvre was defined as the time period during which the ground reaction force was greater than 10 N. The weight acceptance of the stance phase was defined as the interval between foot contact and the first low point in the total ground reaction force (sum of vectors Fx, Fy, and Fz) [32].

The second outcome of this study was the knee flexion angle, which was also evaluated twice in the same manner as the knee valgus angle. The timing of these measurements was based on a previous study that suggested the maximum strain in the ACL is reached at the beginning of stance [33]. Koga et al. [10] reported that ACL damage occurs between initial contact and 40 ms; thus, the time from initial foot contact to peak ground reaction force was evaluated. Two vectors were created along the long axes of the shank and thigh segments and projected onto the global reference planes, and the angles between these vectors were defined as the knee valgus and flexion angles. This method of angle calculation was validated for accuracy against marker-based motion capture data [34–36].

The third evaluation outcome was the varus/valgus moments of the dominant knee at initial contact. To minimize variance and allow comparisons between subjects, the knee joint moments were normalized to body weight and height. Both the shank and thigh segments were modelled as rigid bodies to calculate the moments at each joint centre. Intersegmental moments for each trial were calculated from the joint centre locations, force plate data, and interstitial segment data using an inverse dynamics approach. Next, the centre of mass (COM) was measured, which was the difference between the global positions of the COM and ankle joint centre at initial contact. Each frame was calculated in the sagittal and coronal planes to present a comparative measure of distance of the COM between subjects. The difference in COM in the forward and backward directions was defined as posterior COM, and the difference in COM in the lateral direction was defined as medial COM. In addition, the running speed immediately before foot contact was measured at the speed of the COM of the trunk. The data of these criteria were collected concurrently.

### Data analysis

The data for this statistical analysis were knee valgus and knee flexion angles (measured twice), knee varus/valgus moment at initial contact, posterior and medial COM at initial contact, and the speed of COM. In this study, each criterion was measured multiple times from the same case. When evaluating how foot orthosis affect knee kinematics, it is not appropriate to apply statistical methods such as ANOVA, which assumes that each sample is independent. Therefore, a linear regression analysis was performed, assuming a correlation between the measurements of the same cases in the error term. This analysis is based on the assumption that the differences in foot orthosis height are equally spaced: “low,”1; “mid,” 2; “high,” 3. The types of foot orthoses were used as explanatory variables, and the results of these evaluation criteria were used as objective variables for the analysis. With the “low” foot orthosis as the reference, we investigated how the evaluation criteria changed as the height of the foot orthoses increased. The data were analysed after confirming the shape of the distribution by box-and-whisker plotting and confirming that a normal distribution could be assumed. This regression analysis was performed using SPSS version 12 software (SPSS Inc., Chicago, IL). The level of statistical significance was set at *p* < 0.05. The statistical analysis was selected and conducted by an expert in statistics.

The power analysis of this study was conducted according to a method described by Harrell et al. [37]. They stated that 15 divided n is the number of factors to be corrected in linear regression analysis (n: total number of cases). As the factors can be assumed to be equally spaced in this study, the number of factors was 1; therefore, the results of the power analysis required a minimum of 15 cases.

## Results

The mean values for each evaluation criteria are shown in Table 2, and the results of linear regression analysis are shown in Table 3. The knee valgus angle at initial contact was 2.3 ± 5.2 degrees for “low” medial arch support height, 2.1 ± 5.8 degrees for “mid,” and 0.4 ± 6.6 degrees for “high.” In total, there was a significant correlation between the arch support height of the foot orthoses and the knee valgus angle at initial foot contact in linear regression analysis (*p* = 0.002). The difference between “low” and “mid” was small; however, the knee valgus angle decreased considerably for the “high.” The linear regression formula demonstrated that the knee valgus angle at initial contact significantly decreased more in “mid” than “low” and more in “high” than “mid” medial arch support height. In contrast, there was no significant difference in the knee valgus angle for the peak ground reaction between the height of the foot orthoses. There were no significant differences between the height of the foot orthoses according to items of evaluation that included the knee flexion angle at initial foot contact, knee flexion angle at peak ground reaction, knee varus/valgus moment at initial contact, medial COM, posterior COM, and speed with foot orthosis height.

**Table 2:** Results of evaluation criteria

Kinetic, kinematic, or COM variables	Medial arch support height		
	Low	Mid	High
Knee valgus angle at initial contact (deg) [min, max]	2.3 ± 5.2 [− 11.0, 12.0]	2.1 ± 5.8 [− 12.4, 10.0]	0.4 ± 6.6 [− 11.8, 10.0]
Knee valgus angle at peak contact (deg)^a^ [min, max]	5.2 ± 10.0 [− 10.0, 23.4]	4.7 ± 10.4 [− 10.0, 21.4]	7.4 ± 10.0 [− 10.0, 20.2]
Knee flexion angle at initial contact (deg) [min, max]	11.4 ± 6.6 [0.0, 22.9]	9.6 ± 6.1 [0.0, 25.0]	11.0 ± 6.3 [1.0, 28.0]
Knee flexion angle at peak contact (deg) [min, max]	23.4 ± 9.2 [5.0, 41.0]	22.3 ± 8.0 [8.0, 41.0]	22.5 ± 7.5 [10.0, 39.0]
Knee varus/valgus moment (varus+) (%BW*Ht) [min, max]	2.0 ± 0.7 [0.7, 4.1]	1.9 ± 0.6 [0.3, 3.0]	2.0 ± 0.8 [0.3, 4.2]
Medial distance COM (m) [min, max]	0.26 ± 0.02 [0.21, 0.29]	0.24 ± 0.03 [0.15, 0.31]	0.25 ± 0.03 [0.16, 0.33]
Posterior distance COM (m) [min, max]	0.35 ± 0.05 [0.25, 0.46]	0.35 ± 0.05 [0.26, 0.47]	0.35 ± 0.05 [0.25, 0.47]
Speed (m/s) [min, max]	4.3 ± 0.3 [3.6, 5.0]	4.3 ± 0.3 [3.5, 4.9]	4.3 ± 0.3 [3.7, 5.0]

^a^Negative (−) values indicate varus

**Table 3:** The results of linear regression analysis with the “low” foot orthosis as the reference

		Estimate	Std. error	t value	p value	95% CI (lower)	95% CI (upper)
Knee valgus angle at initial contact (deg)	(Intercept)	− 4.473	1.564	3.534	0.001	2.462	8.593
	Foot orthosis	1.405	0.436	3.223	0.002*	0.550	2.259
Knee valgus angle at peak contact (deg)	(Intercept)	2.002	2.562	4.685	0.000	6.981	17.023
	Foot orthosis	1.633	0.855	1.909	0.059	− 0.043	3.310
Knee flexion angle at initial contact (deg)	(Intercept)	10.790	1.587	6.799	0.000	7.679	13.900
	Foot orthosis	− 0.137	0.439	− 0.311	0.756	− 0.998	0.724
Knee flexion angle at peak contact (deg)	(Intercept)	24.033	2.051	11.717	0.000	20.013	28.053
	Foot orthosis	− 0.605	0.619	− 0.977	0.330	− 1.818	0.608
Knee varus/valgus moment (varus +) (%BW*Ht)	(Intercept)	1.924	0.206	9.358	0.000	1.521	2.327
	Foot orthosis	0.068	0.048	1.420	0.159	− 0.026	0.162
Medial distance COM (m)	(Intercept)	0.255	0.008	33.861	0.000	0.241	0.270
	Foot orthosis	− 0.003	0.003	− 1.075	0.285	− 0.009	0.003
Posterior distance COM (m)	(Intercept)	0.350	0.013	27.449	0.000	0.325	0.375
	Foot orthosis	0.000	0.003	− 0.135	0.893	− 0.007	0.006
Speed (m/s)	(Intercept)	4.325	0.090	48.225	0.000	4.150	4.501
	Foot orthosis	− 0.006	0.026	− 0.233	0.816	− 0.057	0.045

*p < 0.05

## Discussion

The present study demonstrated that the knee valgus angle at initial foot contact can be reduced by the medial arch support of foot orthoses during cutting manoeuvres. In addition, a higher medial arch support resulted in an increased reduction of the knee valgus angle at initial contact. Motion analysis of cutting manoeuvres may be useful in a study of ACL injury mechanisms. To our knowledge, however, there are no previous studies in the literature that have attempted to clarify these injury mechanisms.

Many studies have attempted to improve knee kinematics with the use of foot orthoses. For medial osteoarthritis, the use of a lateral (valgus) wedge orthosis has been reported to reduce the load on the medial compartment and help reduce pain in the medial knee joint [38–40]. Similarly, the use of a medial wedge orthosis can improve symptoms for cases with lateral osteoarthritis [41]. In this study, we speculated that by increasing the longitudinal arch of the foot using the arch support of foot orthoses, the hindfoot valgus angle during cutting manoeuvres would decrease, and the knee valgus angle would decrease accordingly. The results of this study showed that the angle of knee valgus during initial foot contact decreased with the use of foot orthoses with a high medial arch. This may potentially reduce the risk of ACL injury in sports activities caused by knee valgus and reduce the risk of recurrence after postoperative ACL rehabilitation.

The present study demonstrated that foot orthoses reduced the knee valgus angle only at initial foot contact and did not influence the kinematics of the knee joint when the ground reaction force was at its maximum. Therefore, unlike at the moment of initial foot contact, the medial support of foot orthoses may not be effective enough with larger loads. On the other hand, the period between initial foot contact and maximum ground reaction force was around 40 ms in almost all cases in our study. In a study by Koga et al. [10] on the mechanism of ACL injury, ACL tears were reported to occur within 40 ms after initial foot contact. Taking this mechanism into consideration, it is important to understand how to reduce the knee valgus up to 40 ms, and the reduction of knee valgus angle at initial foot contact may be effective in reducing the risk of ACL injury.

To our knowledge, few studies have demonstrated a significant difference of knee kinematics between various types of foot orthoses. Although there have been several studies that examine how foot orthoses affects knee kinematics, it is difficult to detect significant differences in their effects on knee kinematics. Wahmkow et al. [42] evaluated the kinematics of the knee and lower leg when wearing foot orthoses during walking. A comparison of kinematics with and without foot orthoses showed no significant difference between them. The authors suggested that the differences in kinematics per movement between patients were large compared to the small differences in whether a foot orthosis is used, which made it difficult to detect significant differences. Elsewhere, Christopher et al. [43] analysed how knee joint kinematics changed with the use of foot orthoses during landing movements. There was also no significant difference in knee kinematics between those with and without foot orthoses in this study. The authors assessed that several factors were likely to have accounted for the results, including the limitation of a static measure to predict dynamic movement, inter-subject variability, and the physical characteristics of the orthotic device. The absolute value of the knee valgus angle is smaller than that of flexion–extension in a general motion analysis study, and there is a great deal of variation from case to case. Imwalle et al. [44] reported that kinematics of the knee in a cutting manoeuvre showed a large standard deviation of varus and valgus angle. As there is only a small variation in terms of arch support for foot orthoses, finding a significant difference between commercially available foot orthoses would be difficult; therefore, the present study included a foot orthosis with normal height in addition to an overcorrected double-height orthosis.

The results of this study show that the difference in the angle of knee valgus at initial foot contact between orthoses with low and mid medial arch support height is miniscule (0.2° on average). On the other hand, the mean difference between the mid and high arch support height is as large as 1.7°. Based on this result, we found that the foot orthosis with a normal arch support height caused a small reduction in the knee valgus angle, while the foot orthosis with a greater arch support height caused a larger reduction in the knee valgus angle. In general, the foot has a physiological longitudinal arch, and foot orthoses with an arch support of comparable height to physiological height do not have a significant effect on knee kinematics. We believe that foot orthoses with a higher arch support than the physiological height will have a greater impact on the kinematics of the knee.

In the present study, a higher medial arch resulted in a significant reduction of knee valgus angle at initial foot contact. However, foot orthoses with double the normal height were quite uncomfortable for the feet. In fact, some of the subjects complained of discomfort or pain in the medial part of the foot. Su et al. [45] stated that the selection of suitable materials and support designs for orthopaedic foot orthoses may improve the correction of foot arch height but can also result in excessive stress on the joint and ligaments. Foot orthoses should improve the correction of arch height and simultaneously reduce the stress on tissues of the foot; therefore, we believe that orthoses with higher arch support can be worn for a controlled period of time during post-operative ACL rehabilitation but may cause pain and disability in the foot when used for actual sports activities. It remains to be seen what level of arch support will enable the correction of the knee valgus angle without causing foot problems.

Neuromuscular and strength training have been reported to be effective in reducing the risk of ACL injuries [13–19]. While this requires a lot of time and effort, reducing the risk of ACL injury with foot orthoses is a simple, inexpensive, quick, and accessible prophylactic strategy. If foot orthoses are used in conjunction with other treatments such as neuromuscular training, it may be more effective in reducing the risk of ACL injury.

A high valgus knee moment and a small flexion angle at initial foot contact are reported to be risks for ACL injury [12, 46–50]. It has also been reported that a greater distance between the trunk and the centre of the ankle joint (COM distance) at initial foot contact can generate a greater risk [23, 51]. In the current study, these were also assessed at the same time, but there were no significant differences between foot orthoses. The degree to which ACL injury risk is reduced by changing of the knee valgus angle at initial foot contact without changing of valgus knee moment, knee flexion angle, and COM distance was unknown. However, these values have a large inter-individual error with a large variation from one movement to another; therefore, it is difficult to detect significant differences and will require further study in the future.

This study has several limitations. Firstly, the effect of medial arch support on the foot was not evaluated since a motion analysis was not performed around the foot in this study. Because the subjects wore shoes during this study, the presence of shoes made the accurate assessment of the foot difficult to achieve in a motion capture system which we used in this study. Therefore, the kinematics and kinetics of the foot were not assessed. Secondly, the hip joint was not evaluated. As it is not an adjacent joint to the ankle joint, it was determined to be less directly affected by foot orthoses than the knee joint. Thirdly, the subjects of the current study were limited to young female athletes because non-contact ACL injuries are more common in young females. As females have lower muscle density and softer tissues than males, they are expected to be more affected by the arch height of foot orthoses [20, 52–54]. It is unclear whether these results will hold true for male athletes. Fourth, although this study meets the minimum sample size to ensure the accuracy of regression analysis by setting the number of cases, it may be necessary to consider a larger sample size to further improve the accuracy. Fifth, this study did not assess the foot posture scores, hypermobility scores, and range of motion examination of the knee and ankle. Sixth, this study did not assess the height of the arches of individual subjects. In this study, foot orthoses of the same arch height were used for all subjects. It is unclear whether it is effective for people with a high or low arch. Finally, a foot orthosis with double the normal height was quite uncomfortable for the feet and may have affected the measurements. Since this foot orthosis with double the normal height is thought to increase lateral ankle instability, care should be taken when making similar measurements or studies. Although the effect of arch height of subjects warrants further investigation, the findings of this study suggest that medial arch support affects knee joint kinematics during a cutting manoeuvre. Further studies on other motion tasks, male athletes, and individual arch height should be carried out to evaluate the effect of medial arch support of foot orthoses on knee motion.

## Conclusions

This study demonstrated that valgus angle of the knee at initial contact was decreased by the height of the medial arch support provided by foot orthosis during cutting manoeuvres. Medial arch support of foot orthosis may be effective in risk reduction of ACL injury.

## Data Availability

The data that support the findings of this study are available from the corresponding author, W.M., upon reasonable request.

## Abbreviations

ACL: Anterior cruciate ligament
COM: Centre of mass
